# Physiological responses of children with surgically corrected congenital heart disease during high-altitude trekking: an exploratory observational study

**DOI:** 10.3389/fped.2025.1718577

**Published:** 2026-01-12

**Authors:** Kwang Ho Choi, Woong-Han Kim, Ja-Kyoung Yoon

**Affiliations:** 1 Department of Thoracic and Cardiovascular Surgery, Research Institute for Convergence of Biomedical Science and Technology, Pusan National University Yangsan Hospital, Pusan National University School of Medicine, Yangsan-si, Republic of Korea; 2 Department of Thoracic and Cardiovascular Surgery, Seoul National University Children's Hospital, Seoul National University College of Medicine, Seoul, Republic of Korea; 3 JW LEE Center for Global Medicine, Seoul National University College of Medicine, Seoul, Republic of Korea; 4 Department of Pediatrics, Samsung Medical Center, Sungkyunkwan University School of Medicine, Seoul, Republic of Korea

**Keywords:** congenital heart disease, exercise capacity, high altitude, pediatrics, physiological adaptation, surgical correction

## Abstract

**Background:**

Children with surgically corrected congenital heart disease (CHD) are often perceived as fragile and discouraged from engaging in physical activity, despite evidence that structured exercise improves functional capacity and quality of life. However, data on their physiological responses to extreme environments, such as high-altitude exposure, remain limited.

**Methods:**

We conducted an exploratory observational study during an Annapurna Base Camp trek (maximum altitude 4,150 m) in February 2024. Children with surgically corrected congenital heart disease and accompanying healthy adults participated in the expedition. Oxygen saturation (SpO_2_), heart rate (HR), and blood pressure (SBP and DBP) were recorded daily at sequential altitudes, and adverse events were monitored.

**Results:**

From 1,350 m to 4,150 m, SpO_2_ showed a similar pattern of decline in children (−9.8%) and adults (−8.1%). CHD participants showed a pronounced chronotropic response (+17.8 bpm) with modest declines in both SBP (−9.0 mmHg) and DBP (−5.5 mmHg), while adults showed minimal change. These findings suggest effective cardiovascular adjustment under hypobaric hypoxia and with no evidence of maladaptation or altitude-related illness.

**Conclusion:**

Children with surgically corrected CHD showed preserved oxygenation and well-tolerated hemodynamic responses during high-altitude trekking, underscoring their capacity for physiological adaptation when adequately prepared and supervised. The study highlights the feasibility and safety of structured physical challenges for this population and provides preliminary insight into designing exercise programs that promote confidence and participation.

## 1 Introduction

Congenital heart disease (CHD) is the most common birth defect, and advances in surgical and medical care have markedly improved survival, resulting in individuals reaching childhood, adolescence, and adulthood. Nevertheless, many children with CHD continue to face challenges beyond the operating room, particularly regarding exercise capacity and participation in physical activities. Exercise capacity in this population is frequently reported as lower than that of healthy peers, with reductions in peak oxygen uptake, oxygen pulse, and maximal work rates (1, 2). Such limitations may arise from residual anatomical lesions, impaired ventricular function, or reduced capacity for stroke volume augmentation after surgical correction (3).

Beyond physiological constraints, misperceptions of fragility and excessive protection often reinforce inactivity, further compromising physical and psychosocial development (4, 5). Children with CHD are sometimes excluded from physical activity or discouraged from pursuing physically demanding activities, which may contribute to reduced self-confidence and social isolation. Conversely, structured and supervised physical activity has been shown to improve exercise capacity, enhance resilience, and foster social integration (1, 4, 5). And recent randomized evidence indicates that exercise-based rehabilitation can increase overall physical activity levels and improve health-related quality of life in children with CHD (6).

Despite these recognized benefits, little is known about how children with surgically corrected CHD respond to environmental stressors such as high-altitude exposure. Exposure to hypobaric hypoxia provokes characteristic cardiopulmonary adaptation—including hyperventilation, sympathetic activation, tachycardia, and mild pulmonary vasoconstriction—that help maintain oxygen delivery under reduced ambient oxygen tension (7, 8) These responses may vary in children with repaired CHD depending on ventricular reserve, pulmonary vascular reactivity, and residual hemodynamic burden (3, 8). Understanding these mechanisms under hypoxic stress is essential for assessing safety and exercise feasibility in the context of high-altitude trekking in this vulnerable yet capable population.

Furthermore, most children with CHD reside at sea level and have no prior acclimatization experience, raising additional questions about their tolerance to rapid ascent and low-oxygen environments. Clinical data describing such real-world adaptations are scarce.

This study was designed as an exploratory observational analysis rather than a comparative trial. We investigated the physiological responses—oxygen saturation, heart rate, and blood pressure—of children with surgically corrected CHD during a high-altitude trekking expedition to the Annapurna Base Camp. By analyzing individual-level physiological trends of children with CHD during the high-altitude trek, we aimed to describe how these children adapt to hypobaric hypoxia in a real-world setting, providing preliminary insights into the safety and feasibility of such physical challenges.

## 2 Materials and methods

### 2.1 Study design

This was an exploratory observational study based on physiological monitoring data collected during a high-altitude trekking expedition for children with surgically corrected CHD and accompanying adults. Measurements were originally obtained as part of routine safety monitoring and were subsequently analyzed retrospectively.

### 2.2 Participants

The trekking team comprised children with surgically corrected congenital heart disease and accompanying healthy adults, including medical staffs and parents. All participants lived at sea level in Korea and had no prior experience of high-altitude exposure or acclimatization training.

The children had undergone definitive surgical repair 7–12 years before the expedition and were clinically stable in daily life. Although formal pre-expedition cardiology evaluations were not performed for every child, all were known to have no functional limitations in ordinary activities and had undertaken structured physical preparation for approximately one year, hiking nearly every week, including multiple supervised hikes and winter ascents in domestic mountains reaching elevations of around 1,700 m, to improve endurance and adaptation capacity before the expedition.

**Figure 1:**
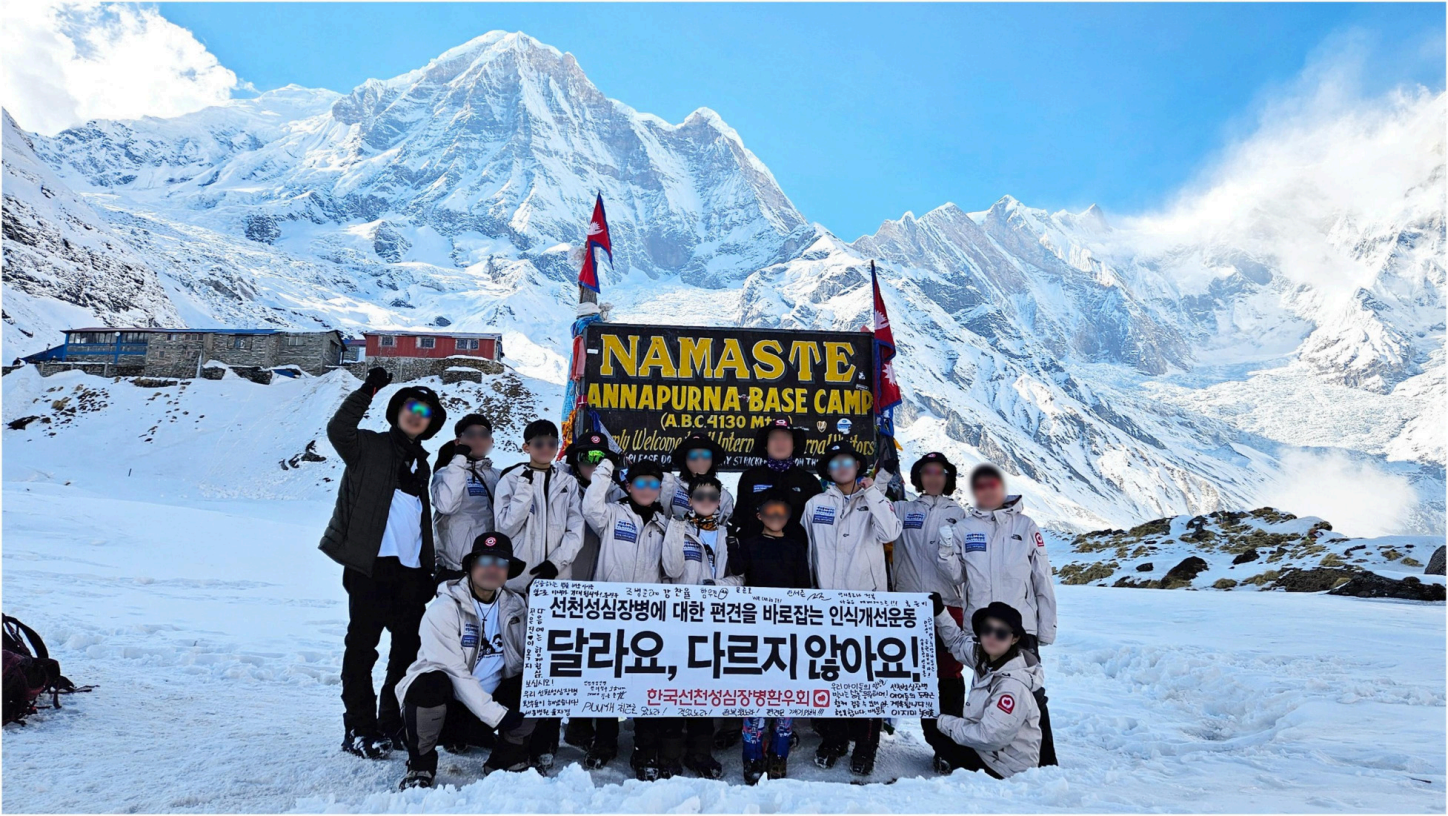
Photograph of the expedition team at Annapurna Base Camp, comprising children with surgically corrected congenital heart disease (CHD), their parents, and medical staff.

The medical staff accompanied the expedition both as participants and as safety supervisors. They were prepared to respond to emergencies and routinely evaluated the physical condition of all participants at each destination. Figure 1 presents a photograph of the expedition team in front of the Annapurna Base Camp sign.

### 2.3 Trekking course

An 11-day expedition to Annapurna Base Camp (maximum elevation 4,150 m) was conducted in Nepal between February 2 and 12, 2024. The course included both ascending and descending segments, rather than a simple upward profile, allowing gradual altitude gain and staged acclimatization.

The itinerary was deliberately designed to include approximately two additional days at intermediate altitudes to allow acclimatization or decent in case of altitude illness. However, no child experienced symptoms requiring schedule modification, and the trek was completed as planned.

### 2.4 Physiological monitoring

Physiological parameters—including oxygen saturation (SpO_2_; %), heart rate [HR; beats per minute (bpm)], and both systolic and diastolic blood pressure (SBP and DBP; mmHg)—were measured at designated elevation levels encountered during the trekking sequence. Participants rested for more than one hour before each measurement to minimize exertional effects. Portable finger-type pulse oximeter (MD300 C22) and an automated oscillometric blood pressure monitor (HEM-7156) were used for non-invasive monitoring. To ensure stabilized measurements after altitude gain, all variables were recorded after more than one hour of rest at each daily destination. Assessments were performed indoors or in sheltered using the same devices throught the expedition, warm environments whenever possible to minimize cold-induced artifacts.

SpO₂ and HR were obtained once after confirming a stable plethysmographic waveform. BP was measured once in the seated position using an appropriately sized cuff. All measurements were performed by three trained medical staff members.

### 2.5 Statistical analysis

Group means and standard deviations were calculated for CHD and control participants at each elevation level. Change (*Δ*) from the baseline (1,350 m) to highest altitude (4,150 m) were computed. Given the small sample size and observational nature of the study, no formal hypothesis testing was performed, and the results are presented descriptively.

For children with CHD, individual trajectories were examined to illustrate inter-individual variability, and adult data were presented separately to demonstrate general altitude-related patterns.

### 2.6 Ethics statement

Measurements were collected for safety monitoring during the trekking expedition and analyzed retrospectively. No additional interventions were performed beyond routine non-invasive monitoring. The requirement for institutional review board approval was waived in accordance with national and institutional regulations, as the study involved retrospective analysis of de-identified safety data. All participants provided written informed consent, with parental consent obtained for children. The study adhered to the principles of the Declaration of Helsinki.

Written informed consent was also obtained from the participants’ legal guardians for the publication of any potentially identifiable images or data included in this article.

## 3 Results

### 3.1 Study population and preparation

A total of fourteen participants, including five boys with surgically corrected congenital heart disease (mean age, 12.8 years) and nine healthy adults (mean age, 47.3 years), successfully completed the 11-day Annapurna Base Camp expedition to a maximum elevation of 4,150 m. No child experienced altitude sickness requiring medical intervention, cardiovascular events or required interruption of the trek.

Individual clinical characteristics—including diagnosis, surgical procedure, time since last surgery, and residual lesions on echocardiographic examination—are summarized in Table 1. The time since the last surgery ranged from 7 to 12 years, and all children were clinically stable in daily life without heart failure symptoms.

**Table 1:** Baseline clinical diagnosis, surgical history, and residual cardiac lesions of children with surgically corrected congenital heart disease.

Patient	Diagnosis	Surgical procedures (type and year)	Time since last surgery (year)	Residual lesions
C-1	PA/IVS	Pulmonary valvotomy and infundibulectomy (2003)	21	Mild to moderate PR and TR
C-2	CoA with ASD	CoA repair (2011)	13	Mild AS
C-3	ccTGA, UVH, DILV	PA banding (2012), Glenn (2013), ECC fontan without fenestration (2015)	9	Mild AR
C-4	DORV with VSD, PS	Initial total correction (2012), Pulmonary valve replacement (2021)	3	Mild to moderate PR, Mild AR
C-5	TGA with VSD	Arterial switch operation (2013)	11	Mild AR

PA/IVS, pulmonary atresia with intact ventricular septum; CoA with ASD, coarctation of aorta with atrial septal defect; ccTGA, congenitally corrected transposition of the great arteries; UVH, univentricular heart; DILV, double inlet left ventricle; DORV with VSD, double outlet right ventricle with ventricular septal defect; PS, pulmonary stenosis; AS, aortic stenosis; AR, aortic regurgitation; PR, pulmonary regurgitation; TR, tricuspid regurgitation; ECC, extracardiac conduit.

### 3.2 Oxygen saturation

**Figure 2:**
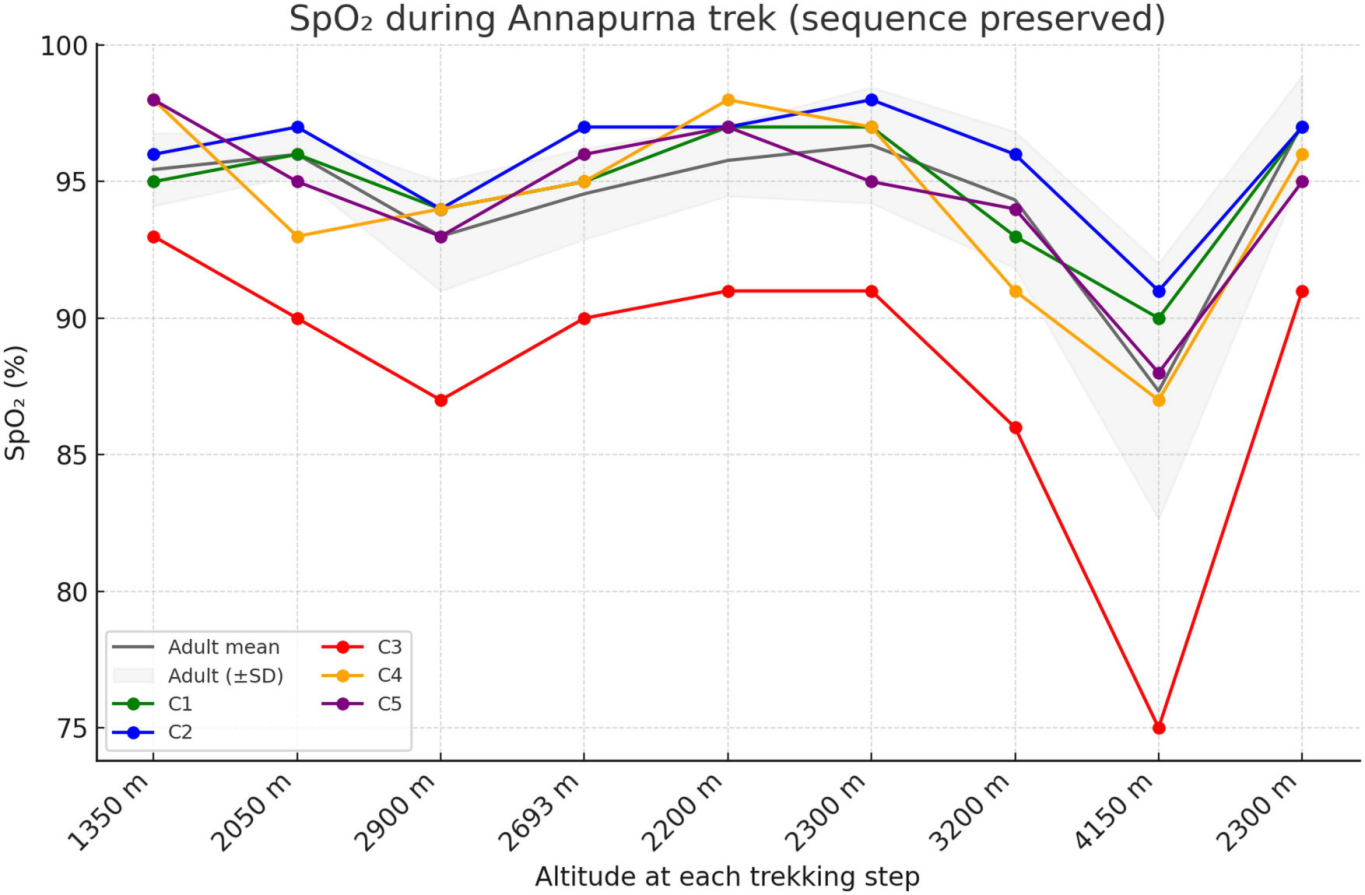
Individual oxygen saturation (SpO_2_) responses during the Annapurna trekking expedition. Oxygen saturation was measured sequentially at each trekking step while maintaining the original ascent and descent sequence. Colored solid line represents individual children with surgically corrected congenital heart disease, and the gray line and shaded area represents the mean ± SD of adult participants.

At 1,350 m, the mean SpO_2_ was 96.0% ± 2.1% in the CHD group and 95.4% ± 1.3% in adults. At 4,150 m, the mean SpO_2_ declined to 86.2% ± 6.5% in children with CHD and 87.3% ± 4.7% in adults (*Δ* −9.8% vs. −8.1%). Individual SpO_2_ trajectories of the five children are displayed in Figure 2, illustrating heterogeneous but well-tolerated desaturation patterns that paralleled the overall trend observed in adults.

However, one child (C3) consistently showed lower absolute SpO_2_ values and a more pronounced decline across altitudes compared with the other participants.

### 3.3 Heart rate

**Figure 3:**
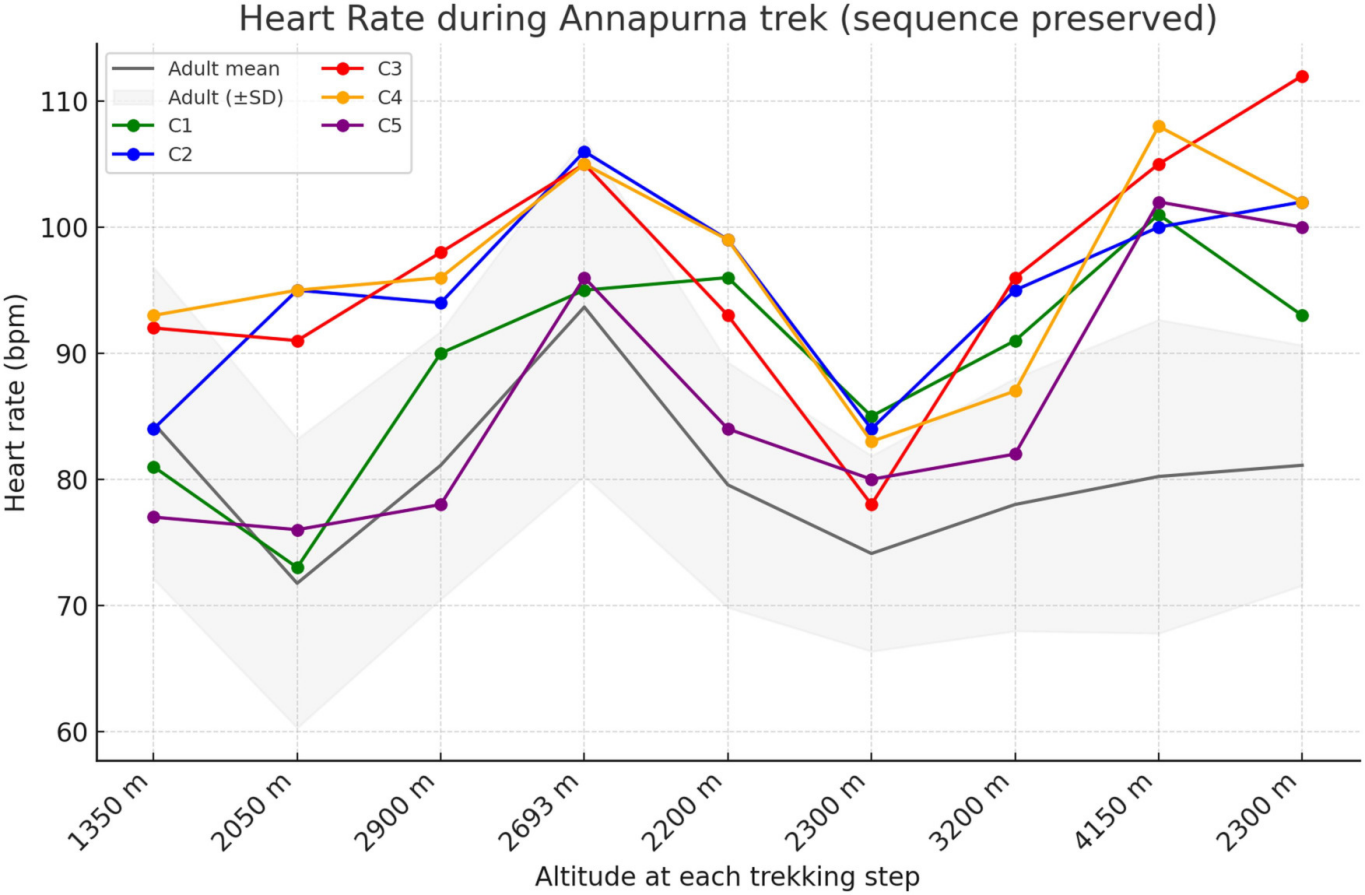
Heart rate responses during the Annapurna trekking expedition. Heart rate changes are shown in sequential trekking order. Colored solid line represents individual children with surgically corrected CHD, while the gray line and shaded area represents the mean ± SD of adult participants.

The mean HR increased from 85.4 ± 6.9 bpm at 1,350 m to 103.2 ± 3.3 bpm at 4,150 m in children with CHD. In contrast, the adults showed a slight decline (84.4 ± 12.4 bpm–80.2 ± 12.4 bpm; *Δ* + 17.8 bpm vs. −4.2 bpm). Individual HR curves (Figure 3) showed a consistent chronotropic response across all children, indicating reliance on heart-rate augmentation to maintain cardiac output at higher altitude.

### 3.4 Blood pressure

Both SBP and DBP were measured and described. SBP decreased from 110.4 ± 18.0 mmHg at 1,350 m to 101.4 ± 14.7 mmHg at 4,150 m in children with CHD, whereas SBP remained similar in adults (116.9 ± 14.1 mmHg vs. 118.4 ± 17.4 mmHg; *Δ* −9.0 mmHg vs. +1.5 mmHg).

DBP showed a modest decline in children with CHD (71.3 ± 11.2 mmHg to 65.8 ± 9.6 mmHg; *Δ* −5.5 mmHg), while adults exhibited relatively little variation (79.4 ± 10.8 mmHg to 77.9 ± 11.6 mmHg; *Δ* −1.5 mmHg).

**Figure 4:**
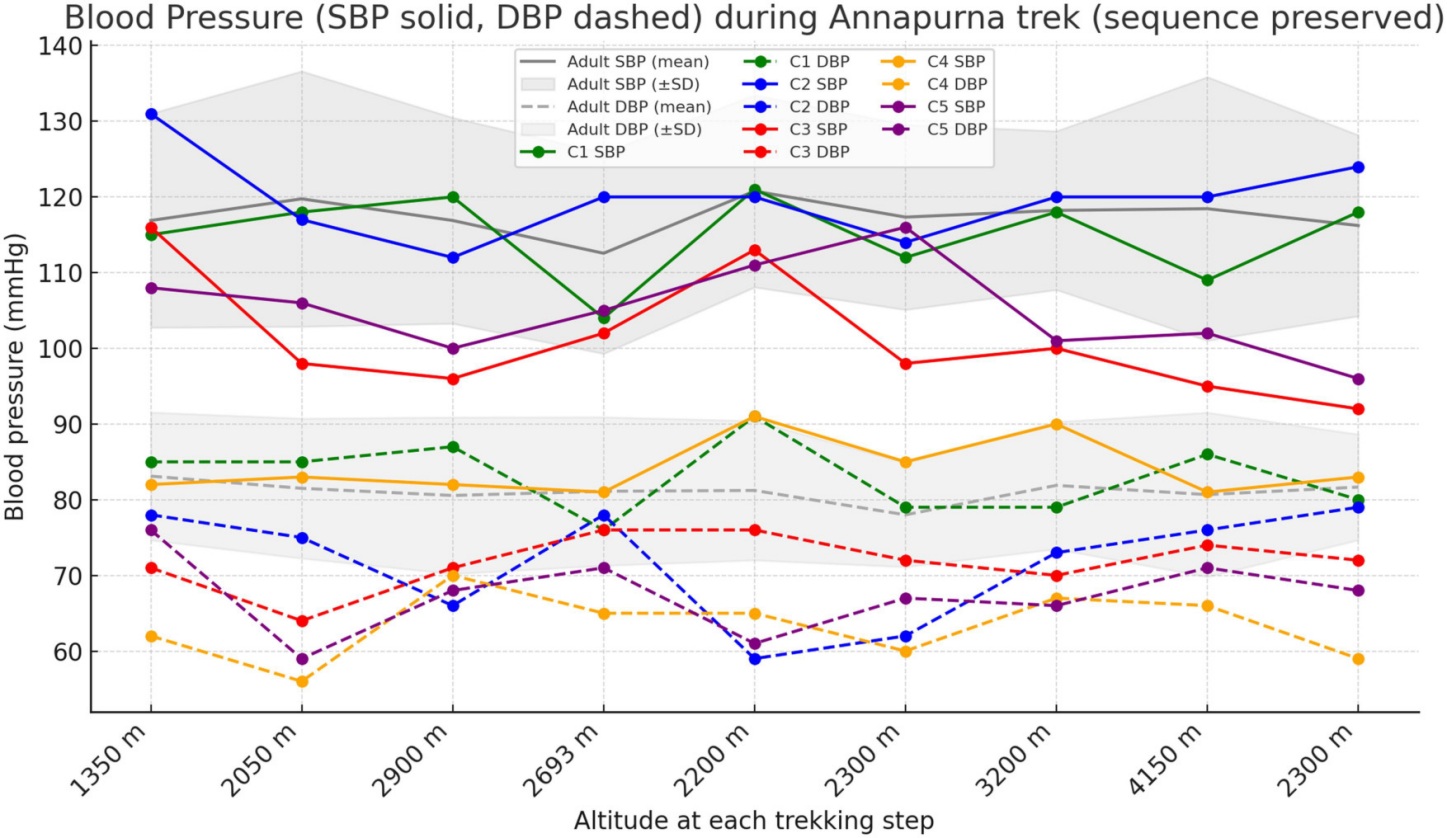
Systolic and diastolic blood pressure responses during the Annapurna trekking expedition. Systolic blood pressure (SBP; solid line) and diastolic blood pressure (DBP; dashed lines) are displayed for each child with surgically corrected CHD. The gray solid and dashed lines represent the adult mean SBP and DBP, respectively, with shaded areas indicating ± SD of adult participants.

Individual SBP and DBP trajectories are shown in Figure 4, demonstrating mild, well-compensated reductions without hypotensive symptoms.

Table 2 summarizes the mean physiological values at baseline (1,350 m) and at the highest altitude (4,150 m) for both groups.

**Table 2:** Changes in oxygen saturation, heart rate, and blood pressure from baseline to the highest altitude in children with surgically corrected congenital heart disease and healthy adults.

		Baseline altitude (1350 m)	Highest altitude (4150 m)	Δ
SpO_2_ (%)	CHD	96.0 ± 2.1	86.2 ± 6.5	−9.8
	Adults	95.4 ± 1.3	87.3 ± 4.7	−8.1
Heart rate (bpm)	CHD	85.4 ± 6.9	103.2 ± 3.3	+17.8
	Adults	84.4 ± 12.4	80.2 ± 12.4	−4.2
Systolic BP (mmHg)	CHD	110.4 ± 18.0	101.4 ± 14.7	−9.0
	Adults	116.9 ± 14.1	118.4 ± 17.4	+1.5
Diastolic BP (mmHg)	CHD	71.3 ± 11.2	65.8 ± 9.6	−5.5
	Adults	79.4 ± 10.8	77.9 ± 11.6	−1.5

***Δ***; values represent the change from baseline (1,350 m) to the highest altitude (4,150 m).

CHD, children with surgically corrected congenital heart disease; SpO_2_, oxygen saturation; BP, blood pressure.

### 3.5 Summary of changes

From the baseline to the highest altitude, both groups showed similar patterns of oxygen desaturation.

Children with CHD showed a more pronounced chronotropic response and modest declines in SBP and DBP, whereas adults showed little variation in cardiovascular parameters. These observations suggest that children with surgically corrected CHD were able to maintain overall hemodynamic stability at high altitude through heart rate augmentation and mild vascular relaxation.

## 4 Discussion

This study shows the feasibility and physiological safety of high-altitude trekking for children with surgically corrected CHD. All participants successfully completed the Annapurna Base Camp expedition without adverse clinical events. The physiological responses observed in this study provide preliminary insight into how this population adapts to hypobaric hypoxia during high-altitude trekking.

Exposure to reduced barometric pressure at altitude triggers characteristic cardiopulmonary responses, including hyperventilation, sympathetic activation, and mild pulmonary vasoconstriction, to maintain oxygen delivery despite ambient oxygen tension (7, 8). In this study, children with CHD showed these expected adaptive responses, suggesting preserved compensatory capacity despite their surgically repaired hearts. Previous exercise studies altitude have similarly shown that hypobaric hypoxia imposes persistent constraints on maximal oxygen uptake and cardiac performance, even after acclimatization (9, 10)

Between 1,350 m and 4,150 m, SpO_2_ decreased similarly in both groups (−9.8% in CHD vs. −8.1% in adults), suggesting that arterial oxygenation was maintained to a similar extent under hypoxic stress. One child (C3) demonstrated lower absolute SpO_2_ values and a slightly larger decline with altitude compared with the other children. This child had univentricular physiology and had undergone extracardiac conduit Fontan operation, and individuals with Fontan circulation are known to have modestly lower baseline arterial oxygen saturation due to passive pulmonary blood flow and mild systemic venous desaturation. These features may contribute to a more pronounced desaturation response under hypobaric hypoxia, while the overall trend remained consistent with the rest of the group.

Children with CHD showed a more pronounced chronotropic response, whereas adults demonstrated little variation in HR. The more pronounced increase in HR observed in the children is consistent with known pediatric cardiovascular physiology, in which cardiac output during physiological stress is achieved predominantly through chronotropic augmentation rather than stroke volume expansion (11).

Children with CHD also exhibited mild decreases in SBP and DBP, whereas adults showed little variation in cardiovascular parameters. These parallel reduction in SBP and DBP may reflect adaptive vasodilation under hypobaric hypoxia, which has been described as part of normal altitude acclimatization (7, 8).

Because altitude-related physiological responses may exhibit delayed components, we acknowledge that dwell times at each elevation were not identical during the trekking itinerary. However, all measurements were performed indoors after more than one hour of rest using same devices and trained personnel, minimizing the influence of delayed physiological adjustment on the recorded values.

Our observations align with previous studies reporting reduced exercise capacity in children and adolescents with CHD compared with healthy peers (2, 4, 12–14), often attributed to limited stroke volume reserve or residual hemodynamic burden (3, 7). The present findings extend these findings by providing real-world physiological data under hypobaric conditions, showing that, when adequately prepared and supervised, children with repaired CHD can exhibit effective cardiopulmonary adaptations even during physical demanding activity.

From a clinical perspective, these results reinforce current AHA and ESC guidelines, which emphasize that most children with CHD can safely engage in appropriately designed physical activity (5, 15). The findings also complement recent randomized evidence demonstrating that structured exercise-based rehabilitation can improve physical activity levels and health-related quality of life in this population (6). Together, this study adds preliminary evidence supporting outdoor and endurance-type programs aimed at improving fitness, confidence, and social participation in this population.

This study has several limitations, which must be acknowledged. The small sample size and heterogeneity of diagnoses limit generalizability. The adult participants were older rather than age-matched, so direct comparisons should be interpreted cautiously. The observational design precludes causal inference. Future work including age-matched cohorts, detailed cardiopulmonary exercise testing, and echocardiographic or hemodynamic monitoring could better characterize altitude adaptation mechanisms in this group. Although all physiological measurements were standardized and obtained indoors after more than one hour of rest to minimize short-term variability, minor differences in dwell time at each altitude were inherent to the trekking itinerary. This residual variation is unlikely to have materially influenced the overall physiological patterns observed. In addition, repeated measurements at each elevation were not feasible in this field setting, and individual differences in physical conditioning may have contributed to subtle variability across participants.

Beyond physiological findings observed during this trek, this study highlights the psychosocial dimensions of living with CHD. Misperceptions of fragility and excessive protection can perpetuate inactivity and social isolation, whereas structured opportunities for physical achievement may foster resilience, and self-efficacy.

The motto of the Korea Congenital Heart Disease Patients Group—“We are Different, We are not Different”—captures this dual reality: although these children are born with unique challenges, proper preparation and support enable them to thrive their peers.

## 5 Conclusion

Children with surgically corrected CHD successfully completed a high-altitude trekking expedition and showed preserved oxygenation and well-compensated cardiovascular responses during trekking. These findings may support the possibility that, when adequately trained and medically supervised, such children are capable of safely participating in physical demanding activities. Future collaborative studies may help establish structured exercise guidelines tailored to this growing population. Beyond physiology, this study highlights the broader message that although children with CHD are born different, with appropriate surgical correction, preparation, and supervision, they are not different in their ability to adapt, endure, and thrive alongside their peers.

## Data Availability

The raw data supporting the conclusions of this article will be made available by the authors, without undue reservation.
